# Generalized Independence in the *q*-Voter Model: How Do Parameters Influence the Phase Transition?

**DOI:** 10.3390/e22010120

**Published:** 2020-01-19

**Authors:** Angelika Abramiuk, Katarzyna Sznajd-Weron

**Affiliations:** 1 Department of Applied Mathematics, Wrocław University of Science and Technology, 50-370 Wrocław, Poland; angelika.abramiuk@gmail.com; 2 Department of Theoretical Physics, Wrocław University of Science and Technology, 50-370 Wrocław, Poland

**Keywords:** opinion dynamics, voter model, phase transitions, noise, scaling

## Abstract

We study the *q*-voter model with flexibility, which allows for describing a broad spectrum of independence from zealots, inflexibility, or stubbornness through noisy voters to self-anticonformity. Analyzing the model within the pair approximation allows us to derive the analytical formula for the critical point, below which an ordered (agreement) phase is stable. We determine the role of flexibility, which can be understood as an amount of variability associated with an independent behavior, as well as the role of the average network degree in shaping the character of the phase transition. We check the existence of the scaling relation, which previously was derived for the Sznajd model. We show that the scaling is universal, in a sense that it does not depend neither on the size of the group of influence nor on the average network degree. Analyzing the model in terms of the rescaled parameter, we determine the critical point, the jump of the order parameter, as well as the width of the hysteresis as a function of the average network degree 〈k〉 and the size of the group of influence *q*.

## 1. Introduction

Independence appears in models of opinion dynamics under various forms and names, including noise [1,2,3,4,5,6,7,8], inflexibility [9,10,11], zealots [12,13,14,15], non-social state [16,17], social temperature [18,19] or just independence [10,20,21,22,23,24,25]. Regardless of the specific form, it introduces into the system some kind of annealed or quenched disorder, which usually competes with the ordering, and simultaneously the most common form of social influence, namely conformity. This competition leads to the order–disorder (agreement–disagreement) phase transition, whose character may depend both on the values of model’s parameters and the structure of an underlying network.

In social psychology, independence means an absence of influence, contrary to conformity and anticonformity, which mean the existence of social influence, positive, and negative, respectively [26]. This definition seems to be intuitively clear, but how can we recognize this type of behavior within social experiments? Imagine the following situation: an individual, who is a subject (target) of an experiment should choose one of two options: *A* or *B*. A group of people (confederates) tries to convince a target to choose option *B* and thus they all point to *B* as a right choice. Despite their recommendation, a target of influence chooses *A*. What is the type of social response observed within this trial? Is it independence or maybe rather anticonformity? It seems that asking a target about the preference (*A* or *B*) before exposing her or him to the group of influence would be helpful, but it doesn’t ultimately solve the problem [26,27]. For this reason, many different descriptive models defining basic types of social response have been introduced.

Despite the existence of many models, independence is almost always defined in the same way, i.e., as no change in individual’s opinion/behavior. Indeed, such a definition makes independence easy to recognize within an experiment. This is also consistent with the idea of zealots, inflexibility, or stubbornness. However, such a definition does not allow for taking into account situations in which a change of opinion or behavior is not caused directly by the group of influence but results from the individual’s own thoughts, mood, past experiences, etc. In order to describe such a behavior, we need at least a two-dimensional model of social response, in which one dimension (variable) would describe the level of external influence, whereas the second one would correspond to the self-influence. The model that will be studied here corresponds directly to one of such models, specifically to the restructured diamond model [28]. It is composed of two orthogonal conceptual dimensions: (1) the vertical dimension, net conformity, which can be considered as a group (external) influence dimension, and (2) the horizontal dimension, net independence, which can be considered as a self (internal) influence dimension.

The idea of the generalized independence that goes in line with the diamond model has been introduced within the Sznajd model in [20] through the parameter *f*, called flexibility, which determines the position on the self-influence axis: f=0 corresponds to independence, whereas f=1 to self-anticonformity [26,27]. It has been shown that stationary results obtained for the Sznajd model scales with *f*.

The *q*-voter model has been studied in the presence of noise that corresponds to f=1/2 [5,21,23,29], as well as zealotry, which corresponds to f=0 [13,30,31], but the general independence has not been yet considered. In this paper, we ask the question of whether the scaling relation found for the Sznajd model also works for the *q*-voter model with arbitrary *q*, i.e., in the case of continuous (that corresponds to q≤5) and discontinuous (that corresponds to q>5) phase transitions. Moreover, within pair approximation, we check if the scaling is valid for graphs with different average degree of nodes 〈k〉. Finally, we systematically analyze the dependence between the main characteristics of the phase transition (including the jump of the order parameter and the width of hysteresis) and model’s, as well as graph’s parameters.

## 2. The *q*-Voter Model with Generalized Independence

The model studied in this paper is a straightforward generalization of the *q*-voter model with independence, proposed originally in [21] and is directly inspired by the self-influence dimension introduced in a diamond model of social response [26,28,32].

As in the original *q*-voter model with independence, we consider a system of *N* voters placed in the vertexes of a certain undirected graph. Each vertex of a graph is occupied by exactly one voter having a binary opinion, which corresponds to two alternatives such as yes/no, agree/disagree, etc. Therefore, following notation introduced in [33], each voter is in one of two states j∈{−1,1} (often denoted by $\left\{↓,↑\right\}$), which changes in time due to the social response (conformity or independence). Voters placed in the vertexes that are directly linked by the edge are called neighbors and only such voters can interact with each other.

In the original version of the model, voters do not posses any individual traits, which corresponds to the so-called situation (annealed) approach [34]. The dynamics of a single update, of a time length Δt=1/N, is defined as follows:

1. Choose one vertex randomly from the discrete uniform distribution U{1,N}.
2. Decide if in a given update a voter placed in the chosen vertex (target voter) behaves independently (with probability *p*) or conforms to others (with probability 1−p), i.e., choose a random number *r* from the uniform distribution U(0,1) and, if r<p, then a target voter behaves independently; otherwise, it conforms to others.
3. In the case of independence, a state of a target voter changes to the opposite one with probability *f*.
4. In case of conformity, choose randomly without repetitions *q* voters out of all neighbors of a target voter; note that, within this version of the model, we can take into account only such graphs for which a minimum degree of a node is not smaller than *q*. If all *q* voters are in the same state, a target voter takes the same state as *q* voters.

Within the original formulation of the model f=1/2, which does not seem to be very realistic [21,23]. Therefore, here we consider an arbitrary value of *f*.

## 3. Results

Thus far, the general *q*-voter model with independence has been studied only for the fixed value of f=1/2 [21,23]. However, we can easily generalize results for arbitrary value of *f*. Following [23,33], we denote the concentration of nodes in state j∈{−1,1} by $c_{j}$, and the concentration of active links by *b*. An active link connects voters with opposite opinions. By $\theta_{j}=b/\left(2c_{j}\right)$, we express the conditional probability of choosing an active link from all possible links on the condition that we have already chosen a node in state *j*. Following calculations presented in [23], we obtain two differential equations that describe the time evolution of the system of an infinite size:

(1) $$\begin{array}{cc} \frac{\partial c}{\partial t} & =-\sum_{j\in \{-1,\;1\}}c_{j}\left[\left(1-p\right)\theta_{j}^{q}+pf\right]j, \end{array}$$

(2) $$\begin{array}{cc} \frac{\partial b}{\partial t} & =\frac{2}{\langle k\rangle }\sum_{j\in \{-1,\;1\}}c_{j}\left[\left(1-p\right)\theta_{j}^{q}\left[\langle k\rangle -2q-2\left(\langle k\rangle -q\right)\theta_{j}\right]+pf\langle k\rangle \left(1-2\theta_{j}\right)\right], \end{array}$$

where 〈k〉 is an average node degree of a considered network and we denote the concentration of voters with a positive opinion by $c\equiv c_{1}$ for simplicity.

An analytical solution of the above equations for an arbitrary value of *q* is impossible, but, as always, analysis of the stationary properties of the system is much simpler and it is the subject of this paper. The natural question that arises here is if the stationary behavior of the system is interesting from a social point of view. There are at least two reasons for which such an analysis is interesting. Firstly, it allows for investigating phase transitions, and determining its type, the width of hysteresis, etc. The latter is particularly interesting because the hysteresis and tipping points are common features of complex social and psychological systems [35,36]. For example, empirical studies suggest that public opinion exhibits both phenomena, which means that it remains seemingly resistant to change (which is related to hysteresis) and then sudden, abrupt shift of opinion can be observed at the tipping point [37,38]. Secondly, indirectly it allows for reproducing dynamical trends: knowing what is the initial value of the average opinion and values of model’s parameters, we can predict to what state the system will evolve, agreement, or rather the stalemate situation. We discuss this issue in Conclusions.

The stationary values of the concentration *c* of vertexes in state 1 as well as the concentration of active links *b* can be obtained from the stationarity condition:

(3) $$\frac{\partial c}{\partial t}=0\;\mathrm{and}\;\frac{\partial b}{\partial t}=0.$$

Inserting Formulas (1) and (2) into the condition of stationarity (3), we obtain:

(4) $$\begin{array}{cc} p & =\frac{\left(1-c\right)\theta_{-1}^{q}-c\theta_{1}^{q}}{\left(1-c\right)\theta_{-1}^{q}-c\theta_{1}^{q}-f\left(\left(1-c\right)-c\right)}, \end{array}$$

(5) $$\begin{array}{cc} b & =2\frac{c\left(1-c\right)\left[{\left(1-c\right)}^{q}-c^{q}\right]-\frac{q}{\langle k\rangle }\left(1-2c\right)\left[c{\left(1-c\right)}^{q}+\left(1-c\right)c^{q}\right]}{{\left(1-c\right)}^{q}-c^{q}-\frac{q}{\langle k\rangle }\left(1-2c\right)\left[{\left(1-c\right)}^{q}+c^{q}\right]}. \end{array}$$

**Figure 1:**
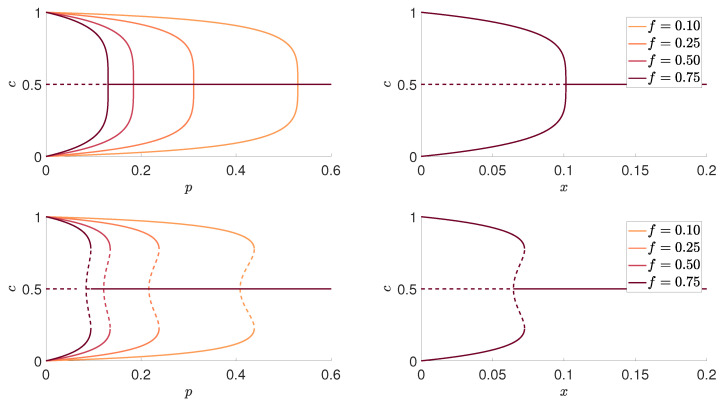
Dependence between the stationary value of the ratio of positive votes *c* and the probability of independence *p* for different values of flexibility *f* for q=5 (upper panels) and q=6 (bottom panels). Original, unscaled results are shown in the left panels, whereas rescaled results, in terms of x=x(p,f), defined by Equation (9), are presented in the right ones. In this example, the average degree of a graph 〈k〉=50. Solid and dashed lines correspond to stable and unstable steady states, respectively.

Flexibility *f* appears only in the formula that relates *p* with *c*, whereas the formula for the density of active bonds (5) is identical as the one derived in [23]. Using the above equations, we can plot the stationary value of positive opinions *c* as a function of independence *p* for arbitrary value of flexibility *f*, as shown in the left panels of Figure 1.

Analogously, as for the original *q*-voter model with independence, there is a phase transition between phases with an order (agreement), i.e., c≠1/2, and disorder (disagreement), i.e., c=1/2. For q≤5, the transition is continuous and therefore a critical point $p^{*}$ separates the phase in which only the ordered state is stable, whereas the disorder is unstable from the phase in which only the disordered state is stable. However, for q>5, the transition becomes discontinuous and thus a critical point $p^{*}$ corresponds to the lower spinodal line. Still, for $p<p^{*}$, only the ordered state is stable, whereas the disorder is unstable. However, above this critical value, the ordered state does not become immediately unstable. Instead, there is an area of metastability, limited by lower and upper spinodal lines, in which both phases can coexist.

Following [23], to derive the analytical formula for the critical point $p^{*}$, we take the limit for c→1/2 using d’Hospital’s rule:

(6) $$\begin{array}{cc} p^{*} & =\lim_{c\to 1/2}p\overset{H}{=}\frac{\left(q-1\right)b^{q}}{\left(q-1\right)b^{q}+f}, \end{array}$$

(7) $$\begin{array}{cc} b^{*} & =\lim_{c\to 1/2}b=\frac{1}{2}\frac{\langle k\rangle -2}{\langle k\rangle -1}. \end{array}$$

As we see, the concentration of active bonds does not depend on flexibility *f* and thus the formula for $b^{*}$ coincides with the one derived in [23]. Inserting *b* in a limit of c→1/2, given by Equation (7) to the Equation (6), we obtain:

(8) $$p^{*}=\frac{q-1}{q-1+2^{q}f{\left(\frac{\langle k\rangle -1}{\langle k\rangle -2}\right)}^{q}}.$$

It is easy to see that, for f=1/2, it reduces to the formula obtained in [23].

As written above, the critical value $p^{*}$ for q>5 corresponds to the lower spinodal. The upper spinodal line cannot be derived analytically, but it can be studied numerically, analogously as for the model on the complete graph [21]. Within such a study, one can investigate how the distance between spinodal lines, i.e., the width of the hysteresis or the jump of the order parameter depends not only on the size of the group of influence *q*, but also on the average degree of a network 〈k〉. Thus far, such systematical studies for the *q*-voter model with independence on random graphs has not been provided even for the f=1/2.

The question that naturally arises here is if there is a real need for introducing an additional parameter *f*. From the psychological point, the parameter *f* describing level of variability is interesting, as it allows for describing a broad spectrum of independent behavior. As is shown in the left panels of Figure 1, agreement is easier for smaller values of *f*, which means that variability does not support an agreement. For example, suppose for a moment that the probability of independence p=0.2 and one community consists of conservative voters (e.g., f=0.1), whereas another community consists of voters with high variability (e.g., f=0.75). In such a case, as visible in the left panels of Figure 1, there will be a high level of agreement, nearly consensus, in the conservative society, but complete disagreement in the non-conservative group.

This result is particularly interesting from the social point of view. Let’s assume for a while that the level of variability, which is a microscopic variable related to the individual behavior (*f* in our model), increased in the modern-day societies. Within our model, it would translate to the decrease of the critical value of *p*, above which there is a stalemate situation, i.e., both opinions are equally likely. In such a stalemate situation, the results of voting on the macroscopic level would be highly unpredictable because random fluctuations could tip the scales of victory to one side.

We do not want to speculate too much on this issue, but it is generally believed that the pace of social change is increasing in many contexts [39]. We are aware that it is far from being clear if the increasing rate of changes on the macroscopic level (results of voting, public opinion, etc.) is caused by the variability in behavior of individuals. Moreover, due to our best knowledge, there are no empirical data directly related to the temporal changes in the degree of variability. However, there are certain premises that could suggest its increment, such as decrease in loyalty to brands [40] or to political parties [41]. To clarify the issue, let us focus on the latter one. In Western democracies, voting behavior has become increasingly volatile over the past decades [42]. A good example illustrating this fact is a steady increase of the proportion of swingers in UK, reported in December 2019 by British Election Study. For instance, in 1966, only about 13% of voters had chosen a different party to the one they supported in the previous election, while, in 2015, 43% of voters have already changed their minds since the previous election in 2010 [41].

**Figure 2:**
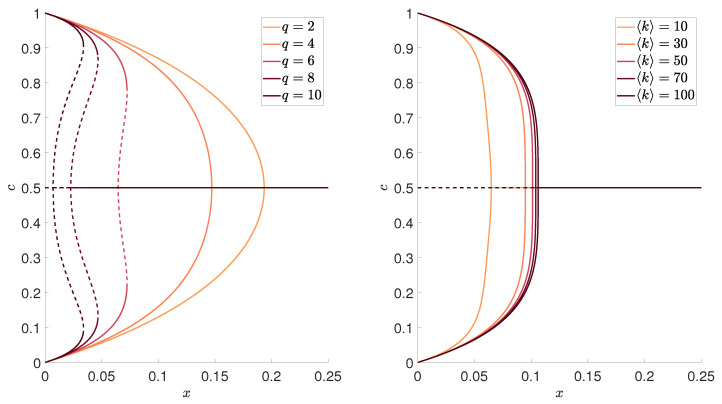
The stationary value of the ratio of positive votes *c* as a function of x=x(p,f), defined by Equation (9), for: different sizes of the group of influence *q* and the fixed average degree 〈k〉=50 (left panel), and different average degree 〈k〉 and the fixed size of the group of influence q=5 (right panel). Solid and dashed lines correspond to stable and unstable steady states, respectively.

On the other hand, flexibility *f* is yet another parameter, which makes a systematic study of the model even more demanding. However, perhaps a scaling relationship could be used to reduce the number of independent variables, which is a common procedure in physics. Indeed, it occurs that scaling derived in [20] for the Sznajd model works also for the *q*-voter model on random graphs for arbitrary value of parameters q,f and 〈k〉; see the right panels of Figure 1. Therefore, if we introduce a new variable:

(9) $$x=\frac{fp}{1-p+fp}$$

and plot results as a function of *x*, instead of *p*, we observe that curves for different *f* collapse to a single one, as shown in the right panel of Figure 1. Therefore, we can represent all results in terms of rescaled variable *x*, defined by Equation (9), as done in Figure 2.

The above scaling can be easily understood, if we notice that we can rewrite Equations (1) and (2) in the form of equations for the original *q*-voter model [23]:

(10) $$\begin{array}{ccc} \frac{\partial c}{\partial t^{\prime }} & = & -\sum_{j\in \{-1,1\}}c_{j}\left[\left(1-p^{\prime }\right)\theta_{j}^{q}+\frac{p^{\prime }}{2}\right]j,\\ \frac{\partial b}{\partial t^{\prime }} & = & \frac{2}{\langle k\rangle }\sum_{j\in \{-1,\;1\}}c_{j}\left[\left(1-p^{\prime }\right)\theta_{j}^{q}\left[\langle k\rangle -2q-2\left(\langle k\rangle -q\right)\theta_{j}\right]+\frac{p^{\prime }}{2}\langle k\rangle \left(1-2\theta_{j}\right)\right], \end{array}$$

with the rescaled time $t\to t^{\prime }=\left(1+\left(2f-1\right)p\right)t$ and independence $p\to p^{\prime }=2pf/\left(1-p+2pf\right)$. It shows that, as far as the steady-state properties are concerned, the model introduced here is equivalent to the standard *q*-voter model with the rescaled independence. Nevertheless, since parameter *f* can be treated as the variable describing self-influence dimension models of social response, we believe that the model presented here is interesting from a social point of view.

As we have already written in the Introduction, we are interested in the dependencies between the main characteristics of the phase transition that appears in this model, and average network degree 〈k〉, as well as the size of the influence group *q*. Such a preliminary analysis has been provided already for f=1/2, but the width of hysteresis and the jump of the order parameter as a function of 〈k〉 and *q* have been never shown. Here, we will present them in terms of the rescaled variable *x*, i.e., for the generalized model with arbitrary value of *f*.

As usual, to describe phase transition, we define an order parameter, as an average opinion at the stationary state, which corresponds to the magnetization in the language of spin models:

(11) $$m=\frac{N_{↑}-N_{↓}}{N},$$

where $N_{↑}$ and $N_{↓}$ denote the stationary numbers of voters with positive and negative opinions, respectively. Because of the stationary concentration of positive voters:

(12) $$c=\frac{N_{↑}}{N},$$

hence there is a simple relation between these two variables:

(13) m=2c−1.

For this reason, they can be used interchangeably. Parameter *c* is usually much more convenient for calculations, but *m* is more appropriate as an order parameter due to its up–down symmetry and can be used for example in the Landau’s approach [21,33].

We start with presenting the dependence between the critical point, below which the agreement, which corresponds to m≠0, is the only stable state:

(14) $$x^{*}=\frac{fp^{*}}{1-p^{*}+fp^{*}}=\frac{q-1}{q-1+2^{q}{\left(\frac{\langle k\rangle -1}{\langle k\rangle -2}\right)}^{q}}.$$

**Figure 3:**
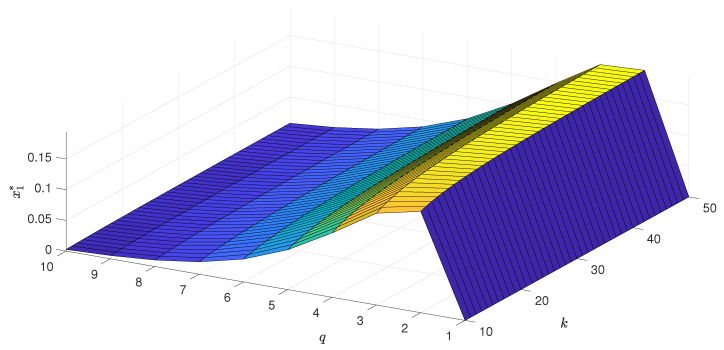
The critical value of the rescaled independence $x_{1}^{*}$ (lower spinodal), below which disagreement is unstable, as a function of the size of the group of influence *q* and the average degree of a graph 〈k〉.

**Figure 4:**
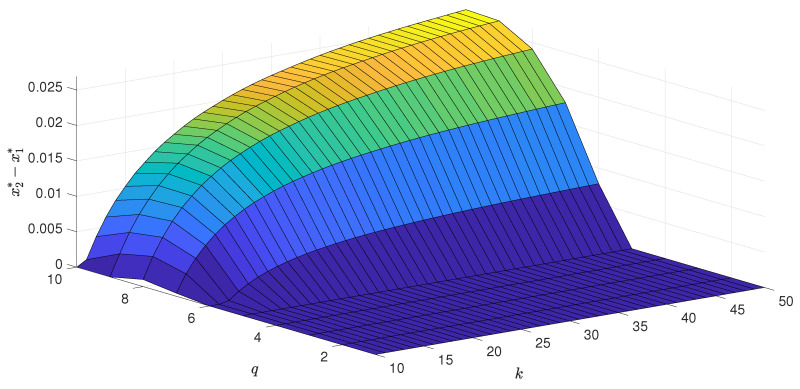
The size of the hysteresis as a function of the size of the group of influence *q* and the average degree of a graph 〈k〉.

**Figure 5:**
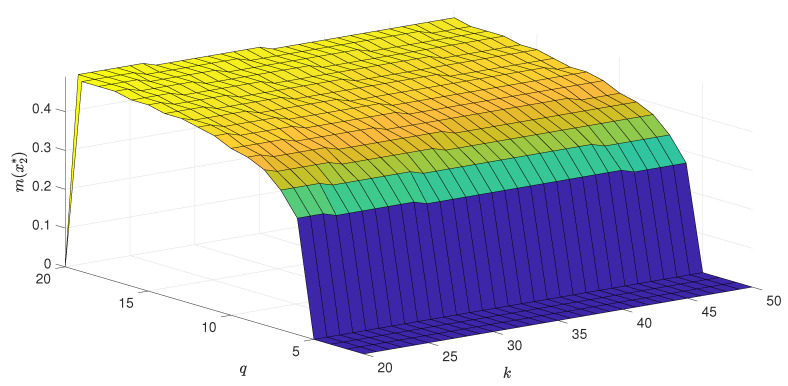
The jump of the public opinion at upper spinodal $x_{2}^{*}$ as a function of the size of the group of influence *q* and the average degree of a graph 〈k〉.

As long as the phase transition is continuous, the only stable state for $x>x^{*}$ is a stalemate situation, which corresponds to m=0. Thus, the system reaches it independently of the initial conditions. However, as we see in the left panel of Figure 2, for q≥6, the transition is discontinuous and hysteresis appears. It means that now *x* corresponds to the lower spinodal line, and let us denote it by $x_{1}^{*}\equiv x^{*}$. Between the lower and upper spinodal line $x_{2}^{*}$, there is region of metastability, in which the final state depends on the initial one. In Figure 3, Figure 4 and Figure 5, we present respectively: the lower spinodal $x_{1}^{*}$, the width of the hysteresis $x_{2}^{*}-x_{1}^{*}$ and the jump of the order parameter *m* at upper spinodal as a function of parameters *q* and 〈k〉. Interestingly, the lower spinodal $x_{1}^{*}$ (see Figure 3) and the jump of the order parameter at the upper spinodal $m(x_{2}^{*})$ (see Figure 5) only slightly depend on 〈k〉. However, the width of the hysteresis $x_{2}^{*}-x_{1}^{*}$ significantly changes with 〈k〉 and generally increases with 〈k〉.

It means that the area of metastability, in which the final state depends on the initial one, increases with the number of neighbors 〈k〉. Intuitively, with the development of the information technologies, such as the Internet, the average number of social links 〈k〉 should increase in the society and this could be responsible for the growth of social hysteresis. Indeed, e.g., the average outdegree of Twitter’s social network has increased over time [43]. However, it is not clear if simultaneously the number of active social contacts has increased [44].Therefore, we can only speculate about the role of 〈k〉 in modern-day societies.

## 4. Conclusions

When building a model, we almost always face the task of choosing the right number of parameters describing a given phenomenon. On one hand, the larger number of parameters usually makes the model more realistic, but, on the other hand, it is not only harder to analyze but also less universal. Therefore, it is not surprising that flexibility *f*, desirable from a psychological point of view and introduced originally for the Sznajd model [20], was a hidden parameter corresponding to f=1/2 in the case of the *q*-voter model.

The reason for which the Sznajd model with independence was studied for different *f*, whereas the *q*-voter model was not is understandable: the *q*-voter model contains one parameter more than the Sznajd model, viz *q* describing the size of the influence group. Moreover, the Sznajd model with flexibility has been studied only on one and two-dimensional lattices, and not on random graphs, which are described by yet another parameter, namely the average node degree 〈k〉. Fortunately, it has occurred that results scale with *f* for arbitrary *q* and 〈k〉, which reduces the number of parameters and allows for introducing a rescaled variable x=x(p,f).

When introducing *x*, we systematically analyze the relationship between different characteristics of phase transitions, including the jump of the order parameter and the width of the hysteresis as a function of *q* and 〈k〉. Such an analysis has never been presented before, even for f=1/2.

The generalization introduced here allows for describing a broad spectrum of independent behavior. Parameter *f*, as introduced here, determines the position on the horizontal (self-influence) axis of the diamond model [26,27,28]. Pure independence (f=0) can be interpreted as self-conformity, which means that targets always agree with her or his initial opinion independently of any social pressures. Similarly, pure self-anticonformity (f=1) means that the target always takes an opposite opinion to the initial one, but, once again, independently of pressure from others. Finally, pure variability (f=0.5) corresponds to the situation in which an individual takes both positions as equally likely (the same as the initial one or the opposite one) [26,45]. It has been shown empirically that self-anticonformity can and does occur. Moreover, the level of *f* can be measured within multi-trial true–false tests; for review, see [26].

We are aware that, in reality, there are individual differences in a way that people respond to the social influence, which has been confirmed empirically, e.g., within experiments on the preference for consistency [46]. Therefore, the level of independence *p*, as well as the level of variability *f* should probably be individual characteristics, instead of being a model’s parameters. Here, we treat them as an average value of individual properties, i.e., as societal characteristics. It is known that such quantities, e.g., individualism/collectivism (I/C) dimension, not only vary across countries, but also change in time. It has been shown that I/C correlates with the level of conformity: more individualistic cultures have lower tendencies to conform; for review, see [46]. Moreover, it has been shown that individualism increases over time [47]. This suggests that the average value independence *p* also increases in time.

Within our model, larger values of *p* indicate a lower level of agreement because the absolute value of public opinion *m* decreases with *p*. However, how exactly it decreases depends on the remaining model’s parameters. For example, with increasing *f*, the critical value of *p*, above which there is a stalemate situation, decreases. It is not clear if indeed *f* increases in time because, to our best knowledge, empirical data that could directly confirm its growth do not exist [45]. Our expectations about the temporal changes of *f* are based only on indirect empirical evidence related to the increasing electoral and consumer volatility [40,42], as discussed in the previous section.

Increasing *f* would make an agreement harder to achieve. This is an interesting result, in the context of empirical data showing that polarization of political opinions is increasing [48,49]. A particularly interesting result relates to roll-call votes cast in the US Congress over the past six decades [48]. Within this study, the result of a vote was coded as 1 for ‘Yes’ and 0 otherwise, and thus the response format was binary like in our model. It was shown that, since the end of 1980s, the polarization between Republicans and Democrats systematically grows. Of course, we cannot link these empirical results directly with our model, since we do not divide the society into two fractions. However, they confirm that disagreement indeed increases in time. What is the reason for that in reality is certainly not obvious. However, since *f* can be measured within a social experiment [26,27], it would be interesting to measure it across different countries and check if it correlates somehow with the polarization of political opinions.

We would like to stress that the generalization introduced here is not the only one, of the *q*-voter model, as reviewed in [33]. For example, the unanimity rule needed for conformity can be replaced by the threshold rule, i.e., conformity takes place if at least $q_{0}$ among *q* neighbors share the same opinion [50,51]. Another point we would like to stress is rather obvious, yet extremely important and often forgotten: the fact that a given model gives realistic results does not mean that this is the only right model for describing opinion dynamics. To validate the model, comparative studies are needed, as shown in [52]. However, we wanted to show that the introduction of a new variable, which seems to be completely irrelevant from the technical point of view (it just rescales results), may be interesting from the social point of view.
